# Assessment of the Antimicrobial Properties of Mesoporous Zinc Oxide Nanoparticles Against *Streptococcus mutans*: An In Vitro Investigation

**DOI:** 10.1155/ijod/4438269

**Published:** 2025-05-06

**Authors:** Zahra Jowkar, Shima Askarzadeh, Seyed Ahmadreza Hamidi, Zahra Fattah, Ali Moaddeli

**Affiliations:** ^1^Oral and Dental Disease Research Center, Department of Operative Dentistry, School of Dentistry, Shiraz University of Medical Sciences, Shiraz, Iran; ^2^Department of Operative Dentistry, School of Dentistry, Shiraz University of Medical Sciences, Shiraz, Iran; ^3^Department of Oral and Maxillofacial Surgery, School of Dentistry, Shiraz University of Medical Sciences, Shiraz, Iran; ^4^Legal Medicine Research Center, Legal Medicine Organization, Tehran, Iran

**Keywords:** antibacterial efficacy, mesoporous nanoparticles, *Streptococcus mutans*, zinc oxide nanoparticles

## Abstract

**Background:** This study focused on synthesizing and characterizing mesoporous zinc oxide nanoparticles (ZnO NPs) while evaluating their antibacterial effectiveness against *Streptococcus mutans*. Their antimicrobial properties were compared to conventional ZnO NPs using minimum inhibitory concentration (MIC) and minimum bactericidal concentration (MBC) tests.

**Methods:** Mesoporous ZnO NPs were produced and analyzed for structural properties. Their antibacterial potential was assessed through MIC and MBC determinations, along with inhibition zone measurements. The test groups included calcined and noncalcined mesoporous ZnO NP solutions (10 mg/mL), standard ZnO NP solution (10 mg/mL), normal saline, and chlorhexidine (CHX) solution (2 mg/mL).

**Results:** All ZnO NP solutions exhibited an MIC of 5 mg/mL, with lower concentrations (2.5 mg/mL and below) showing no inhibition against *S. mutans*. The MIC for CHX (2 mg/mL) was found to be 0.156 mg/mL. MBC values matched MIC results for all NP solutions (5 mg/mL), whereas CHX had an MBC of 0.312 mg/mL. Among the tested solutions, the calcined mesoporous ZnO NP solution produced the largest inhibition zone (19 ± 0.02 mm), followed by the noncalcined version (17.2 ± 0.03 mm). CHX (14.9 ± 0.02 mm) and ZnO NP solution (15.2 ± 0.13 mm) showed similar inhibitory effects.

**Conclusion:** The study suggests that mesoporous ZnO NP solution possesses strong antibacterial properties against *S. mutans*, offering a promising alternative to CHX, which is widely used in dental disinfection. These findings highlight the potential application of mesoporous ZnO NPs in various dental procedures, including endodontics, restorative treatments, and periodontal therapy.

## 1. Introduction

More than half of dental restorations involve replacing previous ones and this trend is steadily increasing [1]. Secondary caries is the leading cause of amalgam restoration replacement, occurring in about 25%–67% of cases, followed by bulk or marginal fractures, which account for 15%–45% [1]. Similarly, secondary caries remain the primary reason for resin composite restoration failure, with occurrence rates ranging from 20% to 44% [1]. Even after removing infected dentin, bacteria can persist on prepared tooth surfaces, contributing to restoration failure and increasing the risk of secondary caries [1]. The presence of residual microorganisms significantly threatens restoration longevity [2]. Among the key bacteria linked to dental caries is *Streptococcus mutans* (*S. mutans*), a highly cariogenic species [2]. Incorporating antibacterial agents into restorative materials or applying an antibacterial irrigant before placing restorations can help prevent bacterial colonization, minimize microleakage, and lower the likelihood of secondary caries [3–5].

Chlorhexidine (CHX) is the most widely used antibacterial cavity disinfectant in dentistry, known for its effectiveness against *S. mutans* and other oral bacteria by disrupting bacterial membrane function [6]. However, CHX has drawbacks, including solubility challenges and a tendency to lose electrostatic interactions with tooth structures, which can reduce its long-term antimicrobial effectiveness [5]. As a result, researchers have focused on developing alternative antibacterial irrigants for dental applications [7, 8].

Zinc oxide (ZnO) is widely utilized in dental materials due to its strong antimicrobial properties [3, 7, 9]. In nanoparticle (NP) form, ZnO demonstrates enhanced antibacterial activity because of its high surface area, stability, biocompatibility, and relatively low toxicity [3, 7, 10]. These NPs combat bacteria by disrupting cell membranes, interfering with enzymatic processes, and generating hydrogen peroxide, which further inhibits bacterial growth [3]. Additionally, ZnO NPs exhibit antioxidant properties and have been classified as safe by the US Food and Drug Administration (21CFR182.8991) [11]. Studies have shown that ZnO NPs can reduce biofilm formation by up to 85% and enhance the antibacterial properties of resin composites against oral bacteria such as *S. mutans* and *Lactobacillus* [4, 12, 13]. However, despite their antibacterial benefits, incorporating ZnO NPs into restorative materials may negatively impact bond strength to tooth structures, making their use as a surface pretreatment a more effective alternative [3, 14]. Recent research highlights their superior antibacterial effects and ability to penetrate dentinal tubules more efficiently than conventional irrigants [15]. ZnO NP colloidal solutions, including those in mouth rinses, have demonstrated effectiveness against *S. mutans* and *Streptococcus sanguis* [16]. In endodontic treatment, ZnO NPs used as a final irrigation solution have been shown to enhance the fracture resistance of treated roots. Additionally, pretreating dentin with ZnO NPs improves its adhesion to glass ionomer cement [7, 8].

Mesoporous materials, defined by pore sizes between 2 and 50 nm, have gained attention in both medical and dental applications due to their high surface area, adjustable pore structures, and biocompatibility [17–20]. Their synthesis allows for modifications in composition, pore size, and structural properties to optimize performance [20]. Among these, mesoporous ZnO NPs stand out for their well-ordered crystalline structure, large surface area, porosity, and potential antibacterial effects [21].

In dentistry, mesoporous materials have been explored for their role in drug delivery and enamel remineralization. Studies have shown that mesoporous calcium–silicate NPs loaded with CHX can promote remineralization, exhibit antibacterial effects against *E. faecalis*, and provide a controlled release of CHX alongside calcium and silicate ions [22]. The incorporation of mesoporous NPs into resin composites has demonstrated strong antimicrobial activity against *S. mutans* and *L. casei* [17]. Furthermore, modifying resin composites with zinc-doped mesoporous silica NPs (Zn-MS NPs) has significantly improved antibacterial effectiveness, with a 15 wt% concentration achieving complete bacterial inhibition [23]. Additionally, previous research has suggested the potential use of mesoporous ZnO NPs in the development of ureteral stents [24].

Due to the limitations of conventional antibacterial irrigants in dentistry, recent research has focused on developing more efficient alternatives [7, 8]. Nanomaterials, particularly ZnO NPs, exhibit enhanced antibacterial properties compared to their bulk counterparts, primarily due to their high surface area-to-volume ratio [25]. With an even larger surface area, mesoporous ZnO NPs have emerged as a promising option for dental antibacterial applications. However, to the best of the authors' knowledge, no study has specifically examined their antibacterial effects against *S. mutans*, the primary bacterium responsible for dental caries. This research aimed to synthesize and characterize mesoporous ZnO NPs, assess their antimicrobial potential against *S. mutans*, and compare their efficacy with conventional ZnO NPs using MIC and MBC assays.

## 2. Materials and Methods

This study was conducted following approval from the Research and Ethics Committee of Shiraz University of Medical Sciences (Protocol # IR.SUMS.DENTAL.REC.1400.027). The synthesis and characterization of mesoporous ZnO NPs were performed following an established procedure [26]. ZnO NP solutions (50 nm particle size) were procured from ASEPE Company, Tabriz, Iran.

### 2.1. Preparation of Mesoporous ZnO NPs

Mesoporous ZnO NPs were synthesized based on a prior protocol with all preparation and calcination steps performed at the Chemistry Department of Shiraz University, Iran [26]. Zinc acetate dihydrate (Zn(Ac)_2_·2H_2_O) was selected as the zinc source, while cetyltrimethylammonium bromide (CTMAB) acted as the structure-directing agent. Both chemicals were obtained from Merck, Germany.

For the synthesis, 1.0 g of CTMAB was dissolved in 480 mL of deionized water under continuous stirring at 80°C to form a uniform solution. Then, 4.92 g of zinc acetate was added and the mixture was stirred thoroughly. The pH was adjusted to an alkaline range using NaOH and the reaction was maintained at 80°C for 2 h. The resulting precipitate was filtered, rinsed with deionized water, and left to air-dry at room temperature for 2 h. The final powder was identified as noncalcined mesoporous ZnO NPs.

The synthesized powder was subjected to heat treatment at 500°C for 4 h using a muffle furnace at the Chemistry Department of Shiraz University. The resulting mesoporous ZnO NPs were kept in a sealed dark plastic bag under dry and cool conditions, away from light, until characterization.

### 2.2. Characterization of Mesoporous ZnO NP

The characterization of mesoporous ZnO NPs followed a previously established procedure [26]. Fourier-transform infrared (FTIR) spectroscopy was carried out at Shiraz University's central lab, while other analyses were conducted at Beam Gostar Taban Laboratory in Tehran, Iran. Particle size, surface morphology, and aggregation were examined using field emission scanning electron microscopy (FESEM) with a TESCAN Mira II (TESCAN, Brno, Czech Republic). For FESEM, samples were placed on conductive stubs, coated with gold, and prepared by dispersing the mesoporous ZnO NP powder in water and drying it at room temperature. Energy dispersive X-ray spectroscopy (EDAX) mapping was used to confirm the elemental composition. X-ray diffraction (XRD) analysis was performed using a Philips PW1730 (Philips, Eindhoven, Netherlands) with Cu–K*α* radiation over a 2*θ* range of 10°–90° at a 0.05° step size and a wavelength of 1.5406 Å, scanning at 0.058°/min. Transmission electron microscopy (TEM) was utilized to analyze the morphology of mesoporous ZnO NPs, with samples placed on a copper grid. Imaging was done with a Philips CM120 microscope (Philips, Eindhoven, Netherlands) at 80 kV. A pellet was created by compressing 2 mg of mesoporous ZnO NPs with 200 mg of FTIR-grade potassium bromide and FTIR spectra were recorded using a Bruker Tensor 27 spectrometer (Bruker Corporation, Billerica, MA, USA) at 4 cm^−1^ resolution [27]. Prior to analysis, mesoporous ZnO NPs were degassed under vacuum at 200°C for 1 h. Nitrogen adsorption/desorption isotherms were measured at 77.3 K using a BELSORP-mini II porosimeter (BEL Japan Inc., Osaka, Japan), with pore size distribution calculated by the Barrett–Joyner–Halenda (BJH) method and surface area determined by the Brunauer–Emmett–Teller (BET) method [28].

### 2.3. Study Groups for Antibacterial Assessments

The study involved evaluating the antibacterial properties of five different solutions. Group 1 consisted of a 10 mg/mL solution of calcined mesoporous ZnO NPs, while Group 2 included a 10 mg/mL solution of noncalcined mesoporous ZnO NPs. Group 3 was a 10 mg/mL solution of standard ZnO NPs. Normal saline was used as the positive control in Group 4, and Group 5 involved a 2 mg/mL solution of CHX, sourced from Ultradent Inc., South Jordan, UT, USA, serving as the negative control. To determine the minimum inhibitory concentration (MIC) and minimum bactericidal concentration (MBC) for each solution, 10 replicates were performed for each concentration.

### 2.4. Determination of MIC, MBC, and Zone of Inhibition (ZOI)

The antimicrobial activity of the test solutions was assessed using the microdilution method in 96-well plates, following a standard broth dilution assay protocol [29].

*Streptococcus mutans* ATCC 25175, sourced from the Iranian Biological Research Center, was used as the bacterial strain. The strain was cultured in nutrient broth (DifcoTM, Detroit, MI, USA) at 37°C with shaking at 250 rpm. Purity checks were regularly carried out to ensure the culture's integrity.

To prepare for the assays, a fresh overnight culture was made by inoculating a loopful of *S. mutans* into nutrient broth and incubating it at 37°C with shaking for 18–24 h. This ensured the bacteria reached the required density and purity was checked to avoid contamination.

For the MIC determination, serial twofold dilutions of 10 mg/mL NP solutions were made in brain heart infusion (BHI) broth, achieving concentrations of 10, 5, 2.5, 1.25, 0.625, 0.0312, and 0.0156 mg/mL. After 24 h, the bacterial suspension was adjusted to a turbidity of 0.5 McFarland standard (1.5 × 10^8^ CFU/mL). Each well received 20 μL of the bacterial suspension and 20 μL of the corresponding study group solution, along with 160 μL of nutrient broth. Control wells were prepared, with positive controls containing broth and test solutions but no bacteria, and negative controls containing inoculated broth without antimicrobial agents.

The MBC was assessed after the MIC test to examine the bactericidal effects of the solutions. For this, 10 μL samples were taken from the wells showing no visible bacterial growth in the MIC test and cultured on nutrient agar. These plates were incubated at 37°C for 24 h. The MBC was identified as the lowest concentration where no bacterial colonies grew on the agar, indicating complete bacterial eradication. This method helped distinguish between bacteriostatic and bactericidal properties of the mesoporous ZnO NPs and other tested groups.

After the 24-h incubation, the inhibition zones around the experimental solutions were measured using a digital micrometer. The ZOI provided an indication of the antimicrobial activity of the solutions against *S. mutans*, with a larger ZOI reflecting stronger antibacterial effects of the NP solution.

### 2.5. Statistical Analysis

The results from the MIC, MBC, and ZOI tests were statistically analyzed. The Kolmogorov–Smirnov test was used to check for normality, and due to nonnormal distribution, the Kruskal–Wallis test was applied, followed by Dunn's post hoc test for pairwise comparisons. Statistical analyses were conducted using SPSS software (version 17, SPSS Inc., Chicago, USA), with significance set at *p* <  0.05.

## 3. Result

### 3.1. Characterization of Mesoporous ZnO NPs

**Figure 1:**
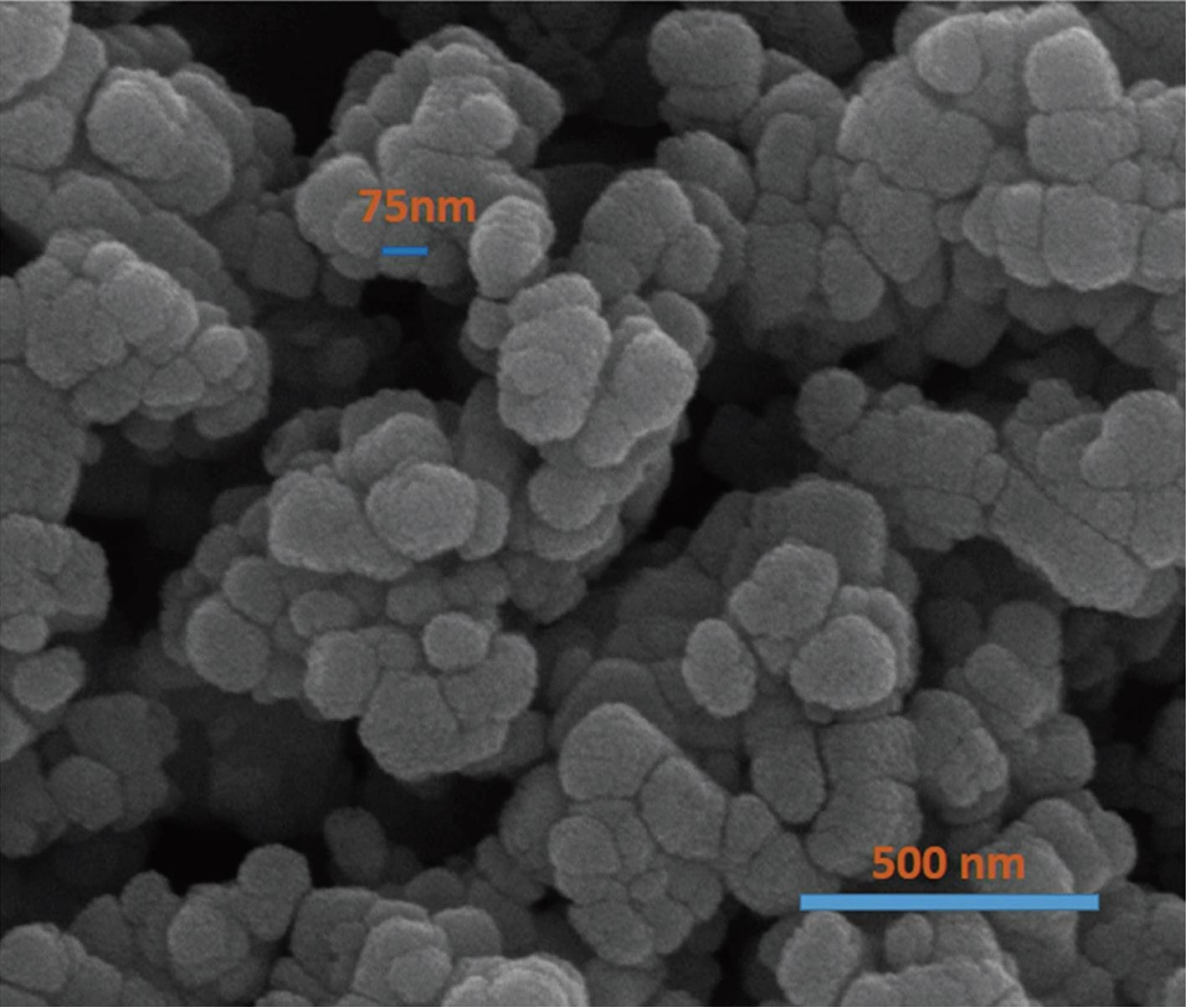
FESEM image of the prepared mesoporous ZnO NPs.

The morphology of mesoporous ZnO NPs was examined using FESEM (Figure 1). The particles primarily displayed a spherical shape with clear grain boundaries and a wide size range of 70–100 nm. They formed consistent aggregates of fine particles, resulting in a dense spherical structure.

**Figure 2:**
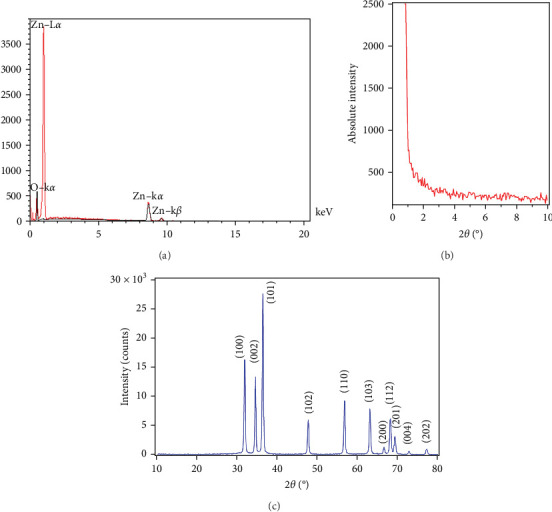
(a) The FESEM EDX elemental mapping of Zn, O, and ZnO. (b) Low-angle XRD pattern of the prepared mesoporous ZnO NPs. (c) Wide-angle XRD pattern of the prepared mesoporous ZnO NPs.

EDX mapping (Figure 2a) demonstrated a uniform distribution of Zn and O elements in the sample.

Low-angle XRD patterns of mesoporous ZnO were recorded between 2*θ* values of 0.85°–10° (Figure 2b), with no distinct characteristic peaks observed in this region.

The XRD patterns of mesoporous ZnO NPs (Figure 2c) displayed prominent peaks at 23.199°, 34.642°, 36.442°, 47.792°, 56.842°, 63.092°, 66.592°, 68.242°, 69.342°, 72.792°, and 77.292°, which are attributed to the [100, 002, 101, 102, 110, 103, 200, 112, 201, 004] and [202] crystal planes, respectively. These peaks confirm the high crystallinity of the ZnO NPs, consistent with standard JCPDS Card No. 036-451.

**Figure 3:**
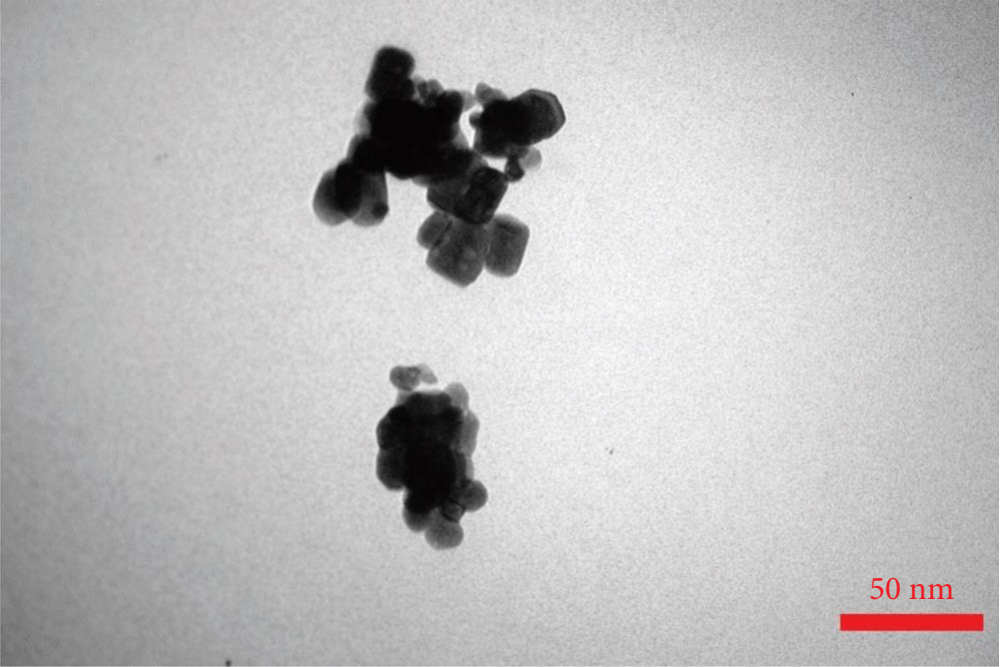
TEM image of the prepared mesoporous ZnO NPs.

**Figure 4:**
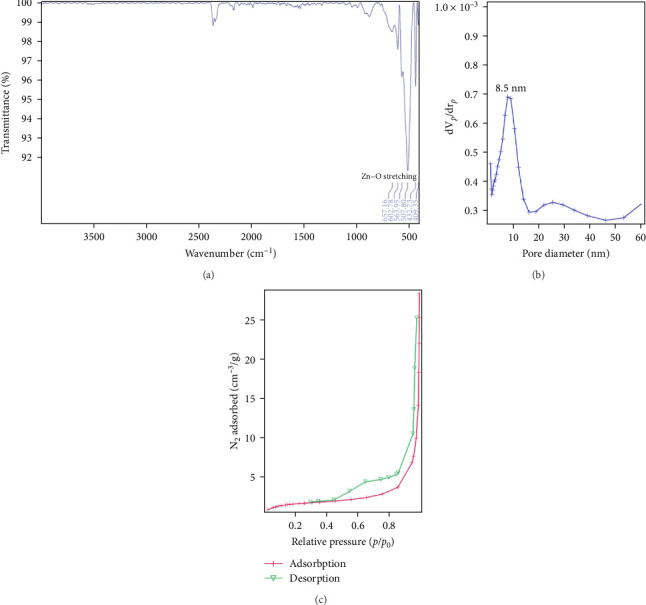
(a) The FTIR spectra of mesoporous ZnO NPs. (b) Pore size distribution plots obtained from the BJH model for the adsorption/desorption branch isotherm of mesoporous ZnO NPs. (c) Nitrogen adsorption–desorption isotherm of the prepared mesoporous ZnO NPs.

TEM imaging was performed to examine the mesoporous structure of the ZnO NPs (Figure 3). The particles were evenly aggregated, with sizes ranging from 10 to 15 nm. A quasi-spherical morphology with surface porosity was observed in many of the particles. Additionally, the NPs clustered together, creating larger pores between them. This characteristic was further confirmed through BJH analysis. FTIR analysis was conducted on the calcined ZnO sample to evaluate its surface composition (Figure 4a). In the ZnO FTIR spectra, an absorption band was observed between 420 and 510 cm^−1^, corresponding to the ZnO transverse optical stretching modes. For mesoporous ZnO NPs, the Zn–O stretching vibration was observed as two distinct peaks at 508 and 433 cm^−1^, verifying the successful formation of ZnO. The mesoporous structure of the ZnO NPs was validated by a Type IV isotherm with an H3 hysteresis loop. BET analysis revealed a specific surface area of 5 m^2^/g. Pore size distribution, assessed by the BJH method (Figure 4b), and N_2_ adsorption/desorption analysis (Figure 4c), showed two predominant pore sizes of 8.5 and 27 nm.

### 3.2. ZOI, MIC, and MBC

**Figure 5:**
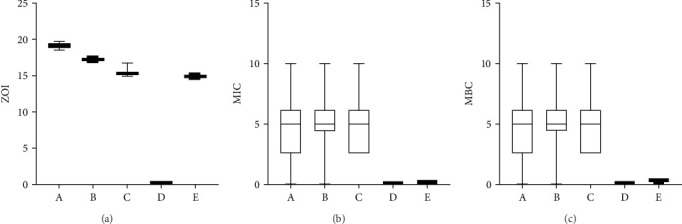
(a) Comparison of the zone of inhibition (ZOI) of the study groups. (b) Comparison of the minimum inhibitory concentrations (MICs) of the study groups. (c) Comparison of the minimum bactericidal concentrations (MBCs) of the study groups. The data of ZOI, MIC, and MBC tests are present as mean ± SD. (A, Group 1: 10 mg/mL calcined mesoporous ZnO NP solution; B, Group 2: 10 mg/mL noncalcined mesoporous ZnO NP solution; C, Group 3: 10 mg/mL ZnO NP solution; D, Group 4: Normal saline; E, Group 5: 2 mg/mL CHX solution).

Figure 5 presents a comparison of the results for ZOI (Figure 5a), MICs (Figure 5b), and MBCs (Figure 5c) across all study groups. The ZOI for each experimental solution against *S. mutans* was measured (Table 1 and Figure 5a). The calcined mesoporous ZnO NP solution exhibited the largest ZOI (19 ± 0.02 mm), followed by the noncalcined mesoporous ZnO NP solution (17.2 ± 0.03 mm). These mesoporous ZnO NPs demonstrated stronger antibacterial effects than the other groups, including the control group and bulk ZnO NPs. CHX, known for its potent antimicrobial activity, showed a ZOI of 14.9 ± 0.02 mm, while the bulk ZnO NP solution presented a similar ZOI of 15.2 ± 0.13 mm. These results suggest that mesoporous ZnO NPs possess antibacterial properties comparable to CHX, indicating their potential as effective antimicrobial agents. Statistical analysis using the Dunn test showed significant differences in the ZOI between normal saline and the other groups (*p* <  0.05). Both the calcined and noncalcined mesoporous ZnO NP solutions had significantly larger ZOIs than the CHX solution. Moreover, the ZOI for the calcined mesoporous ZnO NP solution was considerably higher than that for the ZnO NP solution (*p* <  0.05).

After 24 hours of incubation, the MIC values were recorded, as shown in Table 2. The MIC was defined as the lowest concentration that inhibited *S. mutans* growth. The MIC results for each group are presented in Figure 5b. A 5 mg/mL NP solution was the minimum concentration to effectively prevent bacterial growth. Concentrations below 5 mg/mL (2.5, 1.25, 0.625, 0.0312, and 0.0156 mg/mL) showed no antibacterial activity against *S. mutans*. For 2 mg/mL CHX, the MIC was 0.156 mg/mL. The MBC for both ZnO NP solutions (10 mg/mL) and mesoporous ZnO NP solutions was the same as their MIC (5 mg/mL), while the MBC for CHX was 0.312 mg/mL (Figure 5c). The Dunn test revealed significant differences between the MIC and MBC of normal saline compared to other experimental groups (*p* <  0.05). Furthermore, significant differences were noted between the MIC and MBC of CHX and the calcined and noncalcined mesoporous ZnO NP solutions, as well as the ZnO NP solution (*p* <  0.05). However, no significant difference was found between the MIC and MBC of the mesoporous ZnO NP solutions and ZnO NP solution (*p*  > 0.05).

Figure 5b,c present the MIC and MBC values for the experimental groups. The resemblance between the positive control (normal saline) and the negative control (CHX) in these figures is mainly due to the scale of the *y*-axis and the data distribution in the box plots. As shown in Table 2, CHX (2 mg/mL) had a MIC of 0.156 mg/mL and an MBC of 0.312 mg/mL, indicating its strong antibacterial properties, while normal saline showed no antibacterial effect. The statistical difference between these groups is significant in the table, even though it may not be as apparent in the graphs. A more detailed *y*-axis would have made the scale unnecessarily large, diminishing clarity for the other groups. Thus, it is important to examine both the graphical and tabular data for accurate interpretation.

The results from the MIC and MBC tests confirm that mesoporous ZnO NPs exhibit considerable bactericidal activity against *S. mutans*. Both the MIC and MBC of 5 mg/mL suggest that these NPs not only inhibit bacterial growth but also effectively eradicate *S. mutans* cells, highlighting their potential as an effective antimicrobial agent for *S. mutans*-related infections.

## 4. Discussion

This study investigated the antibacterial properties of mesoporous ZnO NPs compared to ZnO NPs against *S. mutans* through MIC and MBC tests. The results revealed the calcined mesoporous ZnO NP solution exhibited the largest ZOI. Both mesoporous ZnO NP and ZnO NP solutions demonstrated MIC and MBC values of 5 mg/mL for inhibiting *S. mutans* growth.

CHX is commonly used as a cavity disinfectant in restorative dentistry, recognized for its effectiveness against various pathogenic bacteria [30]. However, its antibacterial effects may not be long-lasting, and previous studies have shown that CHX can compromise the bond strength of adhesive systems to tooth structures [31–33]. This has led to ongoing efforts to find alternative disinfectants for dental applications [8].

ZnO NPs have shown potential as a pretreatment to enhance antibacterial properties before restorative procedures. Although the exact mechanisms remain unclear, several possibilities have been suggested [34]. Zn^2+^ ions are thought to disrupt bacterial cell membranes, particularly in gram-positive bacteria like *S. mutans*, by interfering with enzymes responsible for cell wall synthesis. These ions can also enter the cell, leading to damage to internal structures. Additionally, ZnO NPs produce H_2_O_2_, which interacts with membrane components. Other proposed mechanisms include direct physical damage to the cell membrane, disruption of electron transfer, and blockage of membrane channels through interactions with protein and nucleic acid functional groups [34].

Mesoporous ZnO NPs possess unique properties, including a promising antibacterial activity, large surface area, and high crystallinity, making them a strong candidate for use as an antibacterial irrigant in dentistry [24]. This research evaluated their effectiveness against *S. mutans*, a key bacterium involved in dental caries, and compared their performance with that of ZnO NPs using MIC and MBC tests.

The MIC test measures the lowest concentration needed to inhibit bacterial growth [29], while the MBC test determines the concentration required to reduce bacterial viability by ≥99.9%, indicating cell death. MBC serves as a complement to the MIC [29]. This study focused on evaluating the antibacterial properties of different NP solutions against *S. mutans*, a major cariogenic bacterium. This study compared two types of ZnO, namely, 10 mg/mL ZnO NP solution and 10 mg/mL mesoporous ZnO NP solutions, which were further divided into calcined and noncalcined forms. To remove volatile components, the noncalcined mesoporous ZnO NPs were heated to form the calcined variant. The findings showed that both calcined and noncalcined mesoporous ZnO NPs exhibited comparable antibacterial effectiveness at the same concentration. The MIC for both 10 mg/mL calcined and noncalcined mesoporous ZnO NP solutions against *S. mutans* was 5 mg/mL, which was identical to the MIC for the 10 mg/mL ZnO NP solution.

Previous studies have demonstrated the antibacterial properties of ZnO NPs against *S. mutans* [23, 35, 36]. The MIC values observed in this study align with findings from Hernandez-Sierra et al. [36], who reported an average MIC of 4.86 ± 2.71 mg/mL for ZnO NPs against *S. mutans*. Similarly, Bai et al. [23] showed that resin composites containing 5 wt% Zn-MS NPs significantly reduced bacterial growth, with higher concentrations (10 and 15 wt%) nearly eliminating bacterial colonies. Their analysis found antibacterial rates of 33.3%, 96%, and 99.9% for 2, 5, and 10 wt% Zn-MS NPs, respectively, achieving 100% elimination at 15 wt% [23]. In another study, ZnO quantum dots incorporated in adhesive resin displayed antibacterial activity against *S. mutans* without harming pulp fibroblasts [35]. In this study, ZnO and mesoporous ZnO NPs below 5% concentration did not show significant antibacterial effects, which is consistent with previous research, where 1% and 2% ZnO NPs in GICs did not improve antimicrobial properties [37]. However, direct comparisons between this study and earlier works are challenging due to variations in study designs, sample sizes, data collection methods, and statistical approaches.

The calcined mesoporous ZnO NP solution showed the largest ZOI, followed by the noncalcined mesoporous ZnO NP solution, while the ZOI values for CHX and ZnO NP solutions were similar. The larger ZOI observed for calcined mesoporous ZnO NPs is likely due to the higher purity achieved through calcination. The enhanced antibacterial activity of mesoporous ZnO NPs compared to ZnO NP and CHX solutions may be attributed to their smaller particle size, greater surface area, and increased reactivity, which improve interactions with the environment. Additionally, the reduced size of mesoporous ZnO NPs aids in penetrating bacterial cell walls, boosting their antibacterial effect [38]. It has been previously suggested that Zn release from mesoporous NPs is more efficient than from solid ZnO NPs, enhancing their antibacterial properties [23].

The study findings show that the ZOI of the ZnO NP solutions were mainly dependent on their concentrations, consistent with previous research indicating a strong antibacterial effect of ZnO NPs and a clear link between bacterial inhibition and ZnO NP concentration [39]. Solutions with mesoporous ZnO NP and ZnO NP concentrations below 5 mg/mL did not exhibit antibacterial activity against *S. mutans* in this study.

Based on the findings, 5 mg/mL solutions of mesoporous ZnO NPs and ZnO NPs could be effective antibacterial irrigants in dentistry. Notably, the ZOI assessment revealed that mesoporous ZnO NP solutions demonstrated superior antibacterial activity compared to CHX, which is commonly used as an antibacterial irrigant in clinical settings.

This study is the first to assess and compare the MIC and MBC of mesoporous and nonmesoporous ZnO NP solutions against *S. mutans*, offering valuable insights into their potential antimicrobial uses in dentistry. Given their antimicrobial properties, mesoporous ZnO NPs could have future applications in various fields, including endodontics, prosthodontics, implantology, restorative dentistry, and orthodontics.

The results are clinically significant for the development of new antibacterial agents. The mesoporous ZnO NPs were characterized by a unique mesoporous structure, uniform elemental distribution, and high crystallinity, which contribute to their improved antibacterial effects. The MIC and MBC tests showed that a 5 mg/mL mesoporous ZnO NP solution effectively inhibited *S. mutans*, a key cause of dental caries. Additionally, the calcined mesoporous ZnO NPs exhibited the largest ZOI, highlighting their strong antibacterial activity. These findings suggest that mesoporous ZnO NPs could offer a promising alternative to traditional antibacterial agents for preventing and treating dental caries and other infections. Further research is needed to fully assess their therapeutic potential and clinical applications.

This study has a few limitations that must be considered. One important limitation is that the antibacterial activity of mesoporous ZnO NPs was tested at only one concentration (10 mg/mL), chosen based on prior research and its potential clinical relevance. However, exploring a broader range of concentrations could offer a more thorough understanding of the dose-dependent effects. As this study serves as an initial investigation, future studies should test various concentrations to assess their efficacy. Further research should also focus on the antibacterial effects of mesoporous ZnO NPs against other bacterial strains and in different environmental conditions to better evaluate their potential as alternatives to conventional dental antimicrobials. Additionally, since this study was conducted in vitro, its findings may not fully reflect in vivo conditions. The antibacterial effects were measured after 24 h, suggesting the need for long-term studies. Given that dental infections are often polymicrobial and involve biofilms, future research should look into how mesoporous ZnO NPs interact with biofilm-associated bacteria. Another key consideration is that antibacterial irrigants used during dental procedures should not interfere with the bonding of adhesive materials to tooth structures. Since previous studies have shown variations in bond strength with different adhesive systems, it is important to explore how mesoporous ZnO NPs affect the adhesion of resin composites and bonding agents [40]. Future studies should investigate how mesoporous ZnO NPs influence the mechanical properties and long-term bonding strength of dental materials. The effect of these NPs as fillers on both the physical and mechanical characteristics of dental materials also requires further assessment. Additionally, research should explore their ability to interact with tooth surfaces, their antimicrobial properties against various oral bacteria, and their sustained release of Zn^2+^ for prolonged antibacterial action. Finally, attention should be given to evaluating their cytotoxicity, bioavailability, stability, potential discoloration, and cosmetic impact to ensure their safety for clinical use.

## 5. Conclusion

The mesoporous ZnO NP solution demonstrated significant antimicrobial activity against *S. mutans*, suggesting its potential as an alternative to CHX, a widely used antibacterial irrigant in dentistry. Additionally, the MBC for both mesoporous and ZnO NPs was determined to be 5 mg/mL. This study focused on a single concentration (10 mg/mL), providing a basis for future research. It is recommended that subsequent studies explore a broader range of concentrations to further assess the dose-dependent effects and optimize the application of mesoporous ZnO NPs in dental treatments.

## Figures and Tables

**Table 1:** Mean zone of inhibition (mm) ±SD of experimental groups on *Streptococcus mutans*.

Group number	1	2	3	4	5
Group description	10 mg/mL calcined mesoporous ZnO NP	10 mg/mL noncalcined mesoporous ZnO NP	10 mg/mL ZnO NP	Normal saline (positive control)	2 mg/mL chlorhexidine (negative control)
The mean of inhibition zone	19.1 ± 0.02	17.2 ± 0.03	15.2 ± 0.13	0	14.9 ± 0.02

Abbreviation: ZnO NP, zinc oxide nanoparticle.

**Table 2:** Minimum inhibitory concentration (MIC) of experimental groups on *Streptococcus mutans*.

Group number	1	2	3	4	5
Group description	10 mg/mL calcined mesoporous ZnO NP	10 mg/mL noncalcined mesoporous ZnO NP	10 mg/mL ZnO NP	Normal saline (positive control)	2 mg/mL chlorhexidine (negative control)
Growth	−	−	−	+	−

*Note*: − = there is no growth; + = there is growth.

Abbreviation: ZnO NP, zinc oxide nanoparticle.

## Data Availability

The datasets used and/or analyzed during the current study are available from the corresponding author on reasonable request.
